# Aortic Valve Calcium Score by Computed Tomography as an Adjunct to Echocardiographic Assessment—A Review of Clinical Utility and Applications

**DOI:** 10.3390/jimaging9110250

**Published:** 2023-11-15

**Authors:** Isabel G. Scalia, Juan M. Farina, Ratnasari Padang, Clinton E. Jokerst, Milagros Pereyra, Ahmed K. Mahmoud, Tasneem Z. Naqvi, Chieh-Ju Chao, Jae K. Oh, Reza Arsanjani, Chadi Ayoub

**Affiliations:** 1 Department of Cardiovascular Medicine, Mayo Clinic, Phoenix, AZ 85054, USA; scalia.isabel@mayo.edu (I.G.S.);; 2 Department of Cardiovascular Medicine, Mayo Clinic, Rochester, MN 55905, USA; 3 Department of Radiology, Mayo Clinic, Phoenix, AZ 58054, USA

**Keywords:** aortic stenosis, aortic valve calcium score, computed tomography

## Abstract

Aortic valve stenosis (AS) is increasing in prevalence due to the aging population, and severe AS is associated with significant morbidity and mortality. Echocardiography remains the mainstay for the initial detection and diagnosis of AS, as well as for grading of severity. However, there are important subgroups of patients, for example, patients with low-flow low-gradient or paradoxical low-gradient AS, where quantification of severity of AS is challenging by echocardiography and underestimation of severity may delay appropriate management and impart a worse prognosis. Aortic valve calcium score by computed tomography has emerged as a useful clinical diagnostic test that is complimentary to echocardiography, particularly in cases where there may be conflicting data or clinical uncertainty about the degree of AS. In these situations, aortic valve calcium scoring may help re-stratify grading of severity and, therefore, further direct clinical management. This review presents the evolution of aortic valve calcium score by computed tomography, its diagnostic and prognostic value, as well as its utility in clinical care.

## 1. Introduction

Aortic valve stenosis (AS) is a common cause of valvular heart disease with the disease burden rising significantly over the last two to three decades due to the aging population and increasing population longevity related to advances in healthcare. With age being the most important risk factor, prevalence of AS is estimated to be almost 10% in patients older than 80 years [1]. Globally, it is projected that >12 million patients have some degree of AS with more than 100,000 deaths per year related to this disease [2]. As the severity of AS progresses, there is typically an associated symptom and functional burden as well as significant risk for mortality [1].

Effective treatment with valve replacement is currently available once specific indications are met. There are clear clinical, echocardiographic, and imaging criteria that define the indication and timing for valve replacement [3]. There has been substantial research into the pathophysiology underlying AS development, identification of potential agents and therapeutic targets that may slow its progression, enhanced diagnostic methods for evaluation, as well as improvements in valve prosthesis design and materials used to treat severe AS.

Although echocardiography remains the mainstay for the diagnosis and assessment of AS, cardiac computed tomography (CT) for the assessment of aortic valve (AV) calcification has emerged as a useful complementary diagnostic modality. Calculation of an AV calcium score (AVCS) by CT has become an accepted diagnostic tool to detect and help quantify the severity of AS. Its role in assessing severity of AS is particularly helpful in cases with discordant echocardiographic or clinical parameters. This review will canvass potential challenges encountered with standard echocardiographic assessment, discuss the role of AVCS by CT to assess AS severity, and examine the current data supporting its clinical utility in both diagnosis and prognosis of AS.

## 2. Pathophysiology of Aortic Valve Stenosis

**Figure 1:**
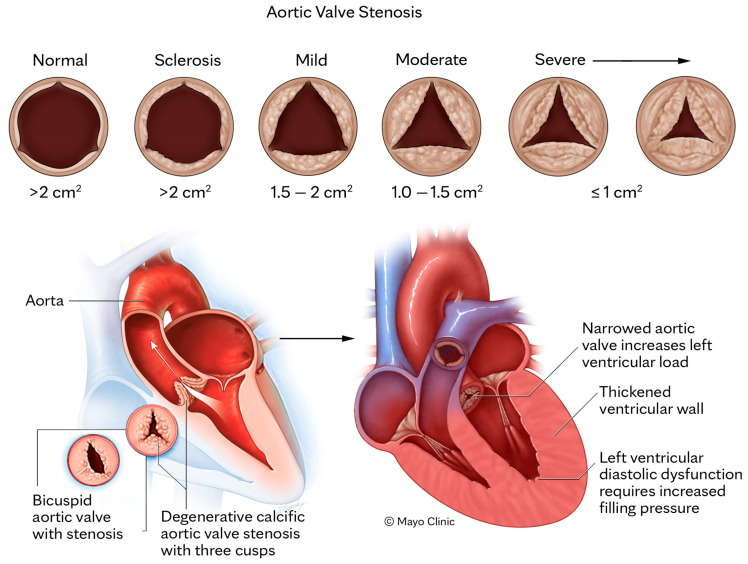
Stylized progression of aortic stenosis in a three-cusp aortic valve with listed areas representing cross-section of aortic valve opening (**top panel**). The bottom left image demonstrates the most common two morphologies in AS, bicuspid valve and three cusp AV with calcific degeneration, and the bottom right panel depicts secondary left ventricular hypertrophy due to severe aortic stenosis (**bottom panel**).

AS has a spectrum of etiologies with bimodal prevalence. Calcific degeneration of the AV is the most common and typically occurs at an older age, whereas bicuspid aortic valve (BAV) and rheumatic valve disease account for AS in younger patients [4]. Overall, the unifying pathophysiologic mechanism is endothelial damage of the valve leaflets leading to inflammation, lipid deposition, fibrosis and thickening, calcification, and ultimately stenosis of the AV (Figure 1) [5]. The increased pressure gradient across the AV due to advancing stenosis leads to maladaptive left ventricular (LV) remodeling, with LV hypertrophy, interstitial fibrosis, myocyte apoptosis and eventual LV failure if severe AS remains untreated (Figure 1).

Clinically, AS can have a prolonged subclinical phase [1]. The disease process starts as aortic sclerosis where there is thickening and focal calcification of the AV cusps without limitation of cusp excursion, and subsequently progressive stenosis develops (Figure 1). Mild or moderate AS typically does not usually affect LV function or cause symptoms, however, progression to severe range AS results in adverse hemodynamic effects on the LV and ultimately development of symptoms; when LV afterload pressure exceeds its ability to compensate. Severe AS typically manifests with symptoms of cardiac failure, syncope, and/or angina [1,6]. Interestingly, despite moderate AS being often asymptomatic, studies have found a significant association between all-cause mortality and moderate AS [7].

Early diagnosis and timely management of AS is imperative, as untreated severe symptomatic AS carries a mortality rate as high as 75% within three years [6]. Currently, there is no known effective medical therapy to prevent progression of AS once it develops. Clinically, patients are monitored until they develop severe or symptomatic AS which indicates the need for invasive intervention either with surgical aortic valve replacement (SAVR) or transcatheter aortic valve replacement (TAVR) [4]. Recent randomized control trials have reported a significant reduction in all-cause mortality, cardiovascular mortality, myocardial infarction, stroke, and heart failure admissions in patients with asymptomatic severe AS who received earlier SAVR, highlighting the importance of early detection, accurate severity grading, and timely referral for treatment of AS, even for asymptomatic patients [8,9,10].

## 3. Echocardiographic Assessment of Aortic Stenosis and Potential Limitations

Transthoracic echocardiography (TTE) remains the backbone for screening, surveillance, and grading of AS severity. Traditional classification of AS has been based on 2D TTE measurements, with current European Society of Cardiology (ESC) and American Heart Association (AHA) guidelines in concordance with cut-off values for severe AS as an aortic valve area (AVA) ≤ 1 cm^2^, AVA indexed to body surface area (AVAi) ≤ 0.6 cm^2^/m^2^, aortic valve mean gradient (MG) ≥ 40 mmHg, and peak aortic velocity (PAV) ≥ 4.0 m/s, in the setting of normal flow across the AV (Table 1) [3,11]. A complementary measurement is the dimensionless index of <0.25 (ratio of left ventricular outflow tract [LVOT] velocity to PAV) [12]. These grading criteria are widely utilized, well established, and have prognostic value [11].

**Figure 2:**
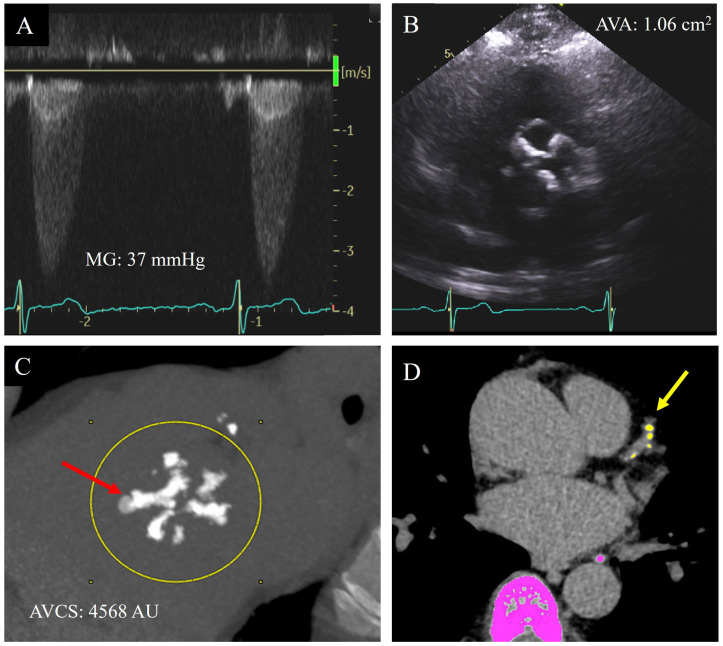
A 69-year-old male with bicuspid aortic valve and shortness of breath on exertion only at high elevations. He had discordant echocardiographic parameters for severity of aortic stenosis, with a clinical echocardiogram report noting overall moderate–severe aortic valve stenosis: systolic mean Doppler gradient (MG) 37 mmHg (**A**), aortic valve area (AVA) by Doppler 1.06 cm^2^ (**B**), dimensionless index 0.23, and normal indexed stroke volume (58 mL/m^2^). He proceeded to have an aortic valve calcium score (AVCS) by cardiac computed tomography ((**C**), red arrow) which demonstrated a score of 4568 AU, reclassifying aortic valve stenosis as severe. This scan also demonstrated calcification in the left anterior descending coronary artery ((**D**), yellow arrow).

There is diagnostic certainty when all quantitative echocardiographic parameters are aligned in either the moderate or severe AS range, corresponding with the visual appearance on 2D echocardiography of limitation of cusp excursion, and match with the presence or absence of symptomology [13]. Differentiation between moderate and severe AS is critical in decision-making for aortic valve replacement. However, this is often complicated in a significant proportion of cases due to discordance between quantitative TTE measurements or with the patient’s symptoms. [11] (Figure 2A,B).

Patient factors such as body habitus or certain disease states, for example, lung hyperinflation secondary to chronic obstructive pulmonary disease, may result in suboptimal TTE image windows and imprecise valvular and hemodynamic measurements [14]. Underestimation of MG and overestimation of AVA (resulting in underestimation of the severity of AS) can occur with suboptimal alignment of the ultrasound probe [15]. Further sources of error may come from the calculation of AVA, which relies on three separate measurements (each being an opportunity for the introduction of error) that are combined in the continuity equation [16]. Moreover, severe calcification and poor echogenicity may contribute to impaired accuracy of LVOT measurement, which is then squared in the continuity equation [17].

A number of studies have reported on the discrepancies between the different hemodynamic components of AS grading. A study by Malouf et al. evaluated a subset of patients with AS in the community setting and identified a discordance between AVA and MG in 67% of patients [18]. Another study by Minners et al. found up to 30% variation in grading of severe AS between the use of AVA vs. MG vs. PAV [19], even in patients with normal LV function. These discrepancies were even greater in patients with decreased left ventricular ejection fraction (LVEF) [19]. Thaden et al. subsequently evaluated the use of Doppler TTE in 100 patients with known severe AS, reporting significant differences in measurements of each hemodynamic value based on the location of the ultrasound probe [15]. They found that up to 23% of patients may be mis-classified if only assessing AS severity via the apical window [15], emphasizing the importance of evaluating measurements at all imaging windows and potential variability with TTE assessment.

**Figure 3:**
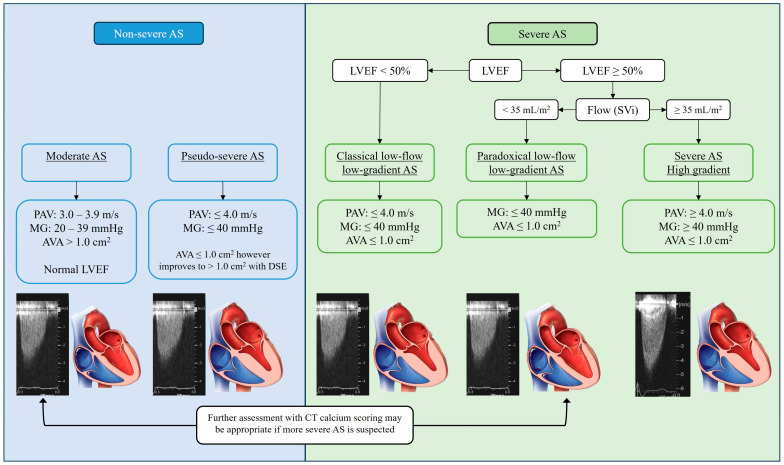
Flow-chart of classification of aortic stenosis (AS) grading based on echocardiographic measurements. Figure has been based off published work with permission from [24]. Copyright 2017 Elsevier. Abbreviations; Aortic stenosis (AS); aortic valve area (AVA); mean gradient across aortic valve (MG); peak velocity across aortic valve (PAV); left ventricular (LV); left ventricular ejection fraction (LVEF); dobutamine stress echocardiography (DSE); stroke volume index (SVi); computed tomography (CT).

Dobutamine stress echocardiography (DSE) may play a role in further evaluation of AS grading in patients where there is uncertainty about AS severity and concern for underestimation, particularly in patients with low LVEF. Such patients have been termed as having “low-flow low-gradient (LFLG) AS”, where abnormalities in LV function or low stroke volume result in lower gradients across a severely stenotic AV (Figure 3). As with resting TTE, DSE carries potential technical limitations given the combination of multiple variables that need to be assessed. Recent studies have questioned the utility of DSE in low-flow AS, reporting poor sensitivity for detecting severe AS and poor correlation with clinical outcomes [20,21]. Several studies suggest that this may be a result of a loss of contractile reserve, making detection of severe AS challenging despite dobutamine load [22,23]. Some conditions associated with LFLG and potential challenges in determining true severity of AS include the presence of cardiac amyloid, atrial fibrillation, concomitant mitral, and tricuspid regurgitation, or varying degrees of obstruction in the LVOT as seen in hypertrophic cardiomyopathy.

Despite such limitations, current guidelines rely heavily on TTE hemodynamics as well as symptomatology for grading of severity and recommendations for invasive therapy in patients with AS. However, uncertainty of AS severity due to discordant TTE findings, in particular in the setting of minimal or unclear symptoms or the presence of confounding comorbidities, can lead to under-referral, under-treatment, or delayed treatment late in disease course and, consequently, poorer outcomes [18]. As such, further evaluation with complementary testing such as with AVCS by CT has been the subject of much research and has been gaining increasing clinical acceptance and utilization.

## 4. Aortic Valve Calcium Score by Computed Tomography

There is a direct pathological link between aortic valve calcification and degree of AS, as calcification is an integral part of the degenerative process [12]. Evaluation of ex-vivo AVs in the early 1990s noted evidence of atherosclerosis, even in very early stages of valve disease [25]. The buildup of calcium deposits on the AV leads to stiffness in opening and decreased valve area. Calcium deposition on AV cusps can occur in varying distributions, which can be visualized on cardiac imaging, including TTE and non-contrast CT. A particular strength of cardiac CT is the detection and quantification of calcification. This is seen in coronary artery calcification (CAC) scoring, which is a well-established and widely used noninvasive method to assess for coronary atherosclerotic burden [26,27].

The initial description of AVCS utilized the Agatston method on cardiac CT, which was initially validated in the 1990s for quantification of CAC (Figure 2C) [17,26,28]. Until 2003, aortic valve calcification had been documented only as an incidental finding on chest CT scans [27,29]. At that time, Cowell et al. assessed 157 patients with contemporaneous Doppler echocardiograms and multi-slice helical CT scans with 2.7 mm slices. This was the first study to address the hypothesis that AV calcification may correlate with hemodynamic markers of AS, confirming a significant relationship between AVCS (measured in Agatston Units, AU) and PAV (r = 0.40; *p* < 0.001), MG (r = 0.54; *p* < 0.0001), and AVA (r = 0.20; *p* < 0.01) [27]. In 2004, Messika-Zeitoun et al. reported a significant correlation (r = 0.96, *p* < 0.0001) between pathological AV calcium weight of explanted valves during SAVR and CT calculated AVCS by the Agatston method [30]. They also evaluated the relationship between AVCS and traditional echocardiographic measurements of AS severity, with a significant curvilinear correlation between AVCS and both AVA (r = 0.79, *p* < 0.0001) and PAV (r = 0.96, *p* < 0.0001).

AVCS has now been well validated in many studies against the hemodynamic TTE markers of AS severity, with the relationships remaining significant when indexed for body surface area as well as age and sex [31]. Ceuff et al. reported a strong correlation between AVCS and AVA (r = −0.63, *p* < 0.0001) [22]. Subsequently, Aggarwal et al. reported a significant relationship between AVCS and AVA indexed to body surface area, MG, and PAV independently (all r > 0.67 and *p* < 0.0001) [14]. Koos et al. further validated AVCS in comparison to invasively obtained hemodynamic measurements with cardiac catheterization, noting a significant relationship with AVA, MG, and PAV (*p* < 0.001 for all measurements) [32].

Numerous studies have since validated the use of non-contrast cardiac electron-beam CT (EBCT), multidetector CT (MDCT), as well as multi-slice CT (MSCT) in calculating AVCS [22,32,33]. Cardiac CT has been shown to reliably and easily quantify AVCS, with low intra- and interobserver variability (3.7 ± 4.3% and 6 ± 7%, respectively) [22,30,34].

## 5. Acquisition and Calculation of AVCS–Standard Protocol

Current standard protocol utilizes non-contrast prospective electrocardiogram-gated CT scans at 120 kVp. The tube current is kept low to reduce radiation exposure, however, may be adjusted to body weight to maintain image quality. Patients do not require intravenous access or contrast, and do not need to be fasted for this scan. The CT scan slice thickness is 2.5 or 3 mm, and calcification is measured at voxel density >130 Hounsfield units (HU), as per the standard Agatston method which is not vendor specific. Areas of calcification outside of the aortic valve (i.e., in the aortic root or proximal coronary arteries) are excluded from the region of interest in each slice. Calcium burden is then quantified by the software by multiplying the area of calcium by a factor related to maximum plaque attenuation: 130–199 HU, factor 1; 200–299 HU, factor 2; 300–399 HU, factor 3; and ≥400 HU, factor 4 [17,26,35,36]. A total calcium score is summated for the aortic valve.

## 6. Prognostic Value of AVCS

Messika-Zeitoun et al. were the first to evaluate for correlation between AVCS and clinical outcomes. Over a two-year follow-up, patients with AVCS ≥ 500 AU had significantly increased AV intervention or cardiac death events compared to those with AVCS below this threshold [30]. Further research has supported this finding, reporting AVCS to be an independent predictor of both all-cause and cardiovascular mortality, even when adjusted for cardiovascular risk factors and coronary artery disease [37,38,39]. The degree of AV calcification predicted up to a four-fold increase in events including death and need for AV surgery [40,41]. This relationship was further demonstrated in findings that AV intervention significantly improved mortality in patients with elevated AVCS compared to no significant improvement in mortality in patients with non-severe AVCS [39].

Similar to AVCS, some studies have evaluated the use of AVCS_density_, calculated as AVCS indexed by aortic annulus area, and have found it to be a more powerful predictor of mortality than AVCS alone [39]. This prediction of events was significant when AVCS_density_ was both concordant and discordant with echo findings [41]. However, in clinical practice the AVCS by the Agatston method is more commonly used over AVCS_density_, allowing for standardized comparability between centers and different scans at different points in time.

## 7. AVCS Quantification Thresholds

Given the increasing evidence for the utility of AVCS in diagnosing AS, as well as predicting associated mortality or need for AV intervention, AVCS cut-offs for determining severe AS have been studied. The initial study by Messika-Zeitoun et al. in 2004 established a threshold for severe AS as ≥1100 AU with a sensitivity of 93% and specificity of 82% [30]. Since then, many studies have sought to validate a clear AVCS threshold for severe AS. A 2021 meta-analysis by Wang et al. assessed subsequent data for AVCS diagnostic utility. They included six studies with significant heterogeneity that examined diagnostic value and ten studies which reported mean AVCS by AS severity, comprising data from 4101 patients [33]. A pooled sensitivity of 82% (95% confidence interval [CI]: 80, 84) and specificity of 78% (95% CI: 75, 81) for AVCS in identification of severe AS was demonstrated, with odds ratio of 15.2 (95% CI: 7.6, 30.4) and an area under the receiver operator curve of 0.87 (95% CI: 0.82, 0.92) [33]. The mean AVCS for severe AS was 3219 AU (95% CI: 2795, 3643), compared to 1252 AU (95% CI: 863, 1640) for those with non-severe AS. Further evaluation of non-severe AS revealed a pooled mean for moderate severity AS to be 1808 AU (95% CI: 1163, 2452) and for mild AS to be 584 AU (95% CI: 309, 859).

## 8. AVCS Differences in Male versus Female

Initially, AVCS thresholds proposed for AS severity did not take sex into account. However, further studies have found male sex to be an independent risk factor for development of AV atherosclerosis [42]. Notably, for the same degree of AS by TTE hemodynamics, women were found to have significantly lower AVCS even when adjusted for age, LVOT and AVA (to account for smaller size and body surface area), and PAV [14]. Physiologically, this has been suggested to be a result of a greater component of fibrosis rather than calcium deposition in the process of valve degeneration in females [43,44].

As such, sex-specific thresholds were proposed for severe AS. Initially, Clavel et al. in 2013 reported a threshold of AVCS ≥ 1274 AU for females, and ≥2065 AU for males for severe AS [31]. This same study identified AVCS_density_ thresholds for severe AS of ≥292 AU/cm^2^ for females, and ≥476 AU/cm^2^ for males. Similar cut-off values for severe AS have since been validated in a large multicenter analysis by Pawade et al. in 2018, with thresholds of ≥1377 AU for females and ≥2062 AU for males [41]. Furthermore, this study highlighted a significant relationship between these determined values and an increased risk of adverse outcomes, specifically AVR and death (hazard ratio 3.90, 95% CI: 2.19, 6.78).

The ESC guidelines have been updated to reflect cut-offs of “very likely severe AS”: males ≥ 3000 AU, females ≥ 1600 AU, and very unlikely severe AS < 1600 AU for males, and <800 AU for females [12]. The AHA guidelines use similar threshold cut-offs of ≥1300 AU for females, ≥2000 AU for males [3]. These sex-specific guidelines are supported by the recent meta-analysis conducted by Wang et al., which found a pooled multivariate hazard ratio of 2.11 (95% CI: 1.11, 4.12) for all-cause mortality associated with AVCS ≥ 1274 AU for females and ≥2062 AU for males in three studies that assessed prognosis [33].

## 9. Progression of AV Calcification

Progression of AS is inevitable, however, the rate of progression is difficult to predict [45]. Early studies into aortic valve calcification have suggested it to be a common process, with more than 25% of patients aged greater than 60 years having some degree of detectable calcification [46]. It was hypothesized that calcification of the AV was likely to have similar pathogenesis to CAC, and, therefore, the risk factors for AV calcification presence and progression would overlap with traditional cardiovascular risk factors [37]. Similarly, the incidence of AV calcification has been shown to be associated with male sex, age, body mass index, smoking, diabetes, hypertension, hyperlipidemia (e.g., lipoprotein(a) level), and use of anti-hypertensive medications [46,47,48]. However, progression of AV calcification and thus AVCS remains an area of ongoing investigation.

Although cardiovascular risk factors have been associated with the presence of AV calcium, only elevated serum low-density lipoprotein (LDL), serum lipoprotein(a) level, and male sex have been found to correspond with AVCS progression over time [37,47,48]. Interestingly, statin therapy has been found to have no impact on progression of AS by both TTE hemodynamics [49] and measurement of AV calcification [50]. Additionally, and independent of cardiovascular risk factors, BAV has been associated with an increased rate of AS progression [51]. Overall, AVCS and the corresponding degree of AS at time of diagnosis have been identified as the most significant determinants of AVCS progression [37,46]. Furthermore, Tastet et al. found that patients with anatomically severe AS, as determined by AVCS and AVCS_density_ at baseline, had a three-fold faster progression of AS hemodynamically (by MG) compared to non-severe AS [52], after adjustment for baseline hemodynamic AS severity and cardiovascular risk factors. This faster progression was also associated with a significant increase in events (death or AV intervention) [52]. There was no significant relationship seen between sex and AS progression [52].

Progression of AVCS was initially quantified by Messika-Zeitoun et al. in 2007, reporting an average AVCS progression of 11 ± 32 AU per year [46]. This study identified a correlation between the presence of AV calcification at baseline and the risk of progression, with 79% of patients with baseline AV calcium having some degree of progression compared to only 10% of patients with no baseline AV calcification. They also showed a relationship between the degree of baseline ACVS and rate of progression, with average annualized AVCS progression of 11 ± 12 AU/year in the lowest tertile baseline AVCS, 20 ± 17 AU/year in the middle tertile, and 86 ± 71 AU/year in the highest tertile. They found no impact of sex on progression, with multivariate analysis confirming the only independent factor associated with progression of AVCS to be baseline AVCS [46].

Subsequently, other studies have evaluated AVCS progression with varying results. Nguyen et al. in 2015 found an average progression of AVCS to be 188 ± 176 AU/year, again reporting a significant correlation between baseline AVCS severity and rate of progression [53]. This study found AV calcium progression in severe AS was 361 ± 293 AU/year, much higher than in previous studies. In comparison to echocardiographic measures, consecutive AVCS measurements over time have been suggested to be superior in detecting small changes in disease progression [35,45].

To summarize these data, a 2023 meta-analysis conducted by Willner et al. found the pooled average annual AVCS progression in AS to be +158.5 AU/year (95% CI: 55.0–261.9 AU), with no significant difference between males and females. In comparison, this meta-analysis found the pooled average yearly progression of MG to be +4.10 mmHg/year (95% CI: 2.80–5.41 mmHg), AVA −0.08 cm^2^/year (95% CI: 0.06–0.10 cm^2^), and PAV +0.19 m/s/year (95% CI: 0.13–0.24 m/s) [54]. Of significance, Willner et al. noted a significant relationship between AVCS, MG, and PAV progression with baseline hemodynamic AS severity, however, AVA progression was not significantly correlated [54].

## 10. Clinical Utility of AVCS by CT

The clinical role and utility of AVCS by CT has evolved as a diagnostic modality that is complementary to traditional echocardiographic assessment of AS. There are several scenarios when AVCS has been found to provide additional information that may re-stratify grading of severity of AS, and, therefore, help direct clinical care and decision on appropriateness of valve replacement. Clinical scenarios where AVCS is particularly helpful include low-flow low-gradient low ejection fraction AS, pseudo-severe aortic stenosis, paradoxical low-flow low-gradient AS, or any situation where there is discordant echocardiographic data, or if AS is suspected to be clinically more significant than the echocardiogram results suggest. Review of AV calcification may also be useful in evaluation/planning for valve replacement, particularly TAVR.

## 11. Low-Flow Low-Gradient Reduced LVEF AS and Pseudo-Severe AS

The first and possibly most frequently utilized setting is that of AS with reduced LVEF, termed “classical low-flow, low-gradient” (LFLG) AS [14,55]. This phenomenon occurs when the AVA is suggested to be ≤1 cm^2^, however, hemodynamic markers of severity (PAV and MG) do not correlate (PAV < 4 m/s and MG < 40 mmHg) [4], likely because the left ventricle is impaired in function and unable to generate the same gradients through a significantly stenosed valve (Figure 3). As such, these patients often have severity of AS under-reported and tend to have poorer clinical outcomes with non-invasive management than high-gradient AS [17,56].

Additionally, TTE is often unable to differentiate LFLG patients from patients with “pseudo-severe AS” characterized by non-severe AS with low LVEF, which gives the appearance of severe AS (Figure 3) [22]. Pseudo-severe AS may be differentiated from severe AS by an improvement in AVA and LVEF with DSE, a change which is not seen in true severe AS [24]. Traditionally, all patients with LFLG or unclear AS severity due to LV systolic dysfunction have been assessed with DSE, however, limitations in the setting of poor contractile reserve decreases the sensitivity of this test, giving rise to the need for complementary assessment as afforded by AVCS by CT [22,23,55].

Accurate assessment of AS is imperative in this patient cohort as they have higher pre-operative risk [57] as well as poorer clinical outcomes, likely due to increased LV myocardial fibrosis as well as systolic and diastolic dysfunction [58]. Similar to traditional high-gradient AS, prognosis is significantly improved with AV intervention [59], and as such, both American and European Guidelines recommend AV replacement in this cohort with a diagnosis of severe AS, regardless of symptoms [3,11]. Notably, women with LFLG AS have a higher mortality risk than men with SAVR and may be better served with TAVR [60,61].

## 12. Paradoxical Low-Gradient AS

Another clinical situation where AVCS has been found to be useful and additive to standard TTE assessment is in paradoxical low-gradient AS, which is defined as AS with low-gradient, low-flow defined by stroke volume index (SVi) < 35 mL/m^2^, and low AVA, but preserved LVEF ≥ 50% (Figure 3) [24]. Hemodynamically, grading of AS severity in this population is challenging and studies have found that approximately half of these patients are graded as severe AS, with the other half being graded as moderate AS [31]. Again, discordance of grading resulting in under-reporting severity of AS may result in a delay in AV intervention and poorer clinical outcomes.

## 13. Clinical Uncertainty or Discordant Data

An additional suggested use for AVCS is as a “tie-breaker” for the management of AS when either the hemodynamic measurements or clinical presentation are unclear or conflicting, creating uncertainty for management decision. Specifically, when severe AS is suspected clinically as a cause of symptoms, however, echocardiographic criteria have not been met to trigger intervention [39,41]. For example, 30–70% of patients with severe AS may present with an MG that is not in the severe range despite AVA < 1 cm^2^ [39]. The severity of AS is difficult to grade in these patients and, therefore, management varies significantly [62,63,64]. In these patients, when stratified by SVi ≤ 35 mL/m^2^, patients with low-flow had significantly worse survival at 3 years (76 ± 4% vs. 86 ± 3%) [65]. Notably, underestimation of severity has resulted in under-treatment of AS which has associated morbidity and mortality [31].

## 14. Pre-TAVR Assessment

TAVR has become increasingly utilized, especially in higher surgical risk candidates or older patients with severe AS [58,66]. Both ESC and AHA guidelines now recognize the use of CT evaluation of AV calcification in the work up of TAVR patients for further quantification and planning of the procedure [3,11]. Bulky eccentric AV calcification has been found to be a significant predictor of paravalvular regurgitation post-TAVR procedure [67,68]. Given that the aortic valve prosthesis is deployed within the diseased native AV in a TAVR procedure, heavy calcification has been associated with increased risk of valve displacement, post-procedure paravalvular regurgitation, and annular rupture as well as coronary ostial obstruction [69]. Thus, a qualitative review of the pattern and degree of calcification on pre-TAVR protocol CT is helpful in procedure planning. Higher AVCS has also been reported as an independent risk factor for acute stroke risk post-TAVR (odds ratio, 1.26; 95% confidence interval, 1.01–1.53; *p* = 0.02) [69,70].

Currently, routine contrast-enhanced CT imaging is performed encompassing the aortic arch to the femoral artery level to assess access in TAVR preparation [69,71]. AVCS quantification by CT typically uses non-contrast images, with studies reporting contrast CT AVCS to be significantly lower than standard non-contrast values, however, there is ongoing research for adjustment factors that may be applied to contrast-enhanced TAVR protocol CT images to estimate an AVCS similar to that derived by the Agatston method [72].

An initial small-scale study by Alqahtani et al. in 2017 suggested a linear relationship for AVCS between non-contrast and contrast-enhanced images with good correlation, proposing the development of an adjustment coefficient [73]. Subsequently, a study by Pandey et al. found a strong linear correlation between AVCS calculated with standard non-contrast AVCS and those derived from ECG-gated contrast-enhanced CT angiography (correlation coefficient, r = 0.9679; *p* < 0.001) [74]. Accordingly, an adjustment formula of AVCS_derived_ = 1.821 × AVCS calculated by ECG-gated contrast-enhanced CT angiography has been proposed, with strong correlation to non-contrast contemporaneous AVCS acquisitions (intraclass correlation coefficient, 0.9648; 95% CI, 0.94–0.98) [74]. As such, it is feasible to derive an estimated AVCS from routine contrast-enhanced CT as part of TAVR workup.

## 15. Asymptomatic Severe AS

Despite conventional indications for AV intervention that include symptoms or LV abnormality associated with severe AS, patients with severe AS who are asymptomatic are also at increased risk of cardiac events or sudden cardiac death [10]. Otto et al. found event-free survival to be only 21% at two years post-diagnosis of severe AS in asymptomatic patients [75]. Another more recent study by Pellikka et al. found only 25% of asymptomatic patients remained free of death or AVR at five years post-diagnosis of severe AS [76]. Notably, in multivariate analysis, there was a significant protective effect of AVR on all-cause mortality [76]. These studies highlight the clinical importance of early diagnosis of severe AS in asymptomatic patients. As such, opportunistic detection of aortic valve calcification on routine nongated CT chest may help with earlier detection of such patients who may benefit from surveillance and consideration for potential treatment. There is ongoing research to help automate AVCS calculation for nongated CT chest which may assist with opportunistic diagnosis.

## 16. Association with CAD

Given the overlap in risk factors for the incidence of AV calcification and CAC, detection of AV calcification may be a marker of coronary artery disease (CAD) (Figure 2D). Studies have found AV calcification to be a marker of subclinical CAD, with a degree of CAD being detected in up to 70% of patients with severe AS [4,58]. Furthermore, the presence of AV calcification is not only associated with a positive CAC score but also correlated with the severity of CAD. Significantly, AVCS has been identified as independent of cardiovascular risk factor for CAD [46].

## 17. AVCS and BAV

**Figure 4:**
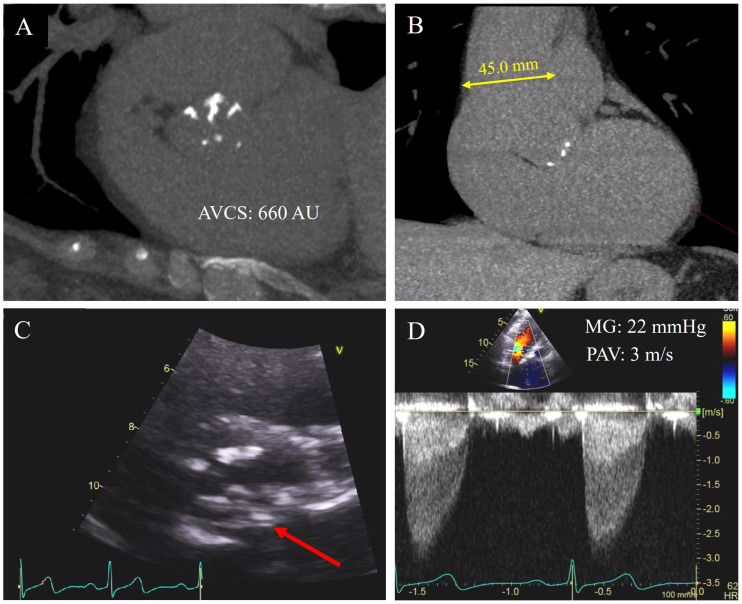
A 62-year-old asymptomatic female sent for coronary artery calcium (CAC) score for risk stratification for statin therapy. The CAC score was 0, but significant aortic valve calcification was identified (**A**). AVCS was quantified as 660 AU. She was also incidentally noted to have mid ascending aorta dilation with diameter of 45 mm (**B**). Subsequent echocardiogram for further evaluation confirmed a bicuspid aortic valve with raphe between the left and non-coronary cusp, demonstrated by arrow (**C**), and overall moderate aortic stenosis with mean gradient (MG) of 22 mmHg and peak aortic velocity (PAV) of 3 m/s (**D**).

BAV is the most common congenital heart disease, with a prevalence as high as 2% in the general population [4]. In these patients, the most common clinical sequela is AS, with up to 37% of patients developing at least moderate AS in their lifetime [3]. Importantly, patients with BAV often develop AS much earlier than those with a tricuspid aortic valve (TAV), presenting up to 15 years earlier [77]. Pathologically, this is thought to be due to higher shear stress on the valve leaflets resulting in earlier fibrosis and calcification [35]. BAV thus leads to preferential calcification of the aortic valve cusps, which progresses to AS and/or aortic regurgitation (Figure 4A,C) [78]. In addition, 20–40% of patients with BAV develop aortic root dilation, associated with an increased risk of aortic dissection (Figure 4B) [3]. Traditionally, patients with severe AS and BAV have been treated with SAVR, however, enhancement in prosthesis design may potentially expand the use of TAVR in BAV. To date, there has been limitation in the use of TAVR in these patients due to the complex anatomy leading to increased post-procedure complications including paravalvular leak and aortic root rupture, which are associated with increased mortality [3,79]. As such, evaluation of AV calcification in BAV patients may allow for better risk stratification and decision-making in the pre-intervention stage.

Initial work suggested that those with BAV may have a lower cut-off to define severe range aortic stenosis, however, subsequent studies have suggested thresholds for severe AS in BAV to be similar to those with severe degenerative AS with three cusps. A 2016 study by Ren et al. investigated AVCS for AS severity in 101 BAV patients, suggesting a threshold for severe AS at 897 AU (sensitivity 86.7%, specificity 72.2%). Interestingly, this is much lower than the previously identified cut-offs for TAV patients [77], despite paradoxically reporting a higher mean AVCS in BAV with severe AS at 3497.71 ± 2470.17 AU. Contrasting with this study, Wanchaitanawong et al. identified an AVCS cut-off in BAV patients (*n* = 43) to be much higher than that noted by Ren et al., with cut-offs closer to those with severe AS with three cusps; AVCS ≥ 1145 AU (sensitivity 83.3%, specificity 80%) for females and ≥2431.8 AU (sensitivity 92.9%, specificity 71.4%) for males [78].

A larger study by Shen et al. in 2022 aimed to clarify this further, assessing 485 patients with BAV [80]. They reported an optimal threshold for severe AS in those with BAV to be ≥2315 AU (sensitivity 82%, specificity 78%) for males and ≥1103 AU (sensitivity 80%, specificity 82%) for females. This study also evaluated the use of previously determined AVCS thresholds for severe AS, specifically those in the original work by Clavel et al. (≥1274 AU for females, and ≥2065 AU for males) and found good accuracy (>80% correct classification) in the detection of AS in their BAV patient cohort [31,80].

## 18. Potential Limitations of AVCS

AVCS has several limitations in its utility of diagnosis, grading, and prognosis of AS. Firstly, dedicated non-contrast CT scans for cardiac calcium scoring carry a small radiation burden of approximately 1 mSv, and not exceeding 3 mSv [81,82]. However, newer reconstructive algorithms are being developed that may reduce the radiation burden to <0.5 mSv per scan [83]. Although this dose is low, repeated scans to monitor progression may need to be applied judiciously to minimize radiation exposure. There is currently no consensus to guide frequency of surveillance AVCS scans if the initial scan is not in the severe range, and presently this continues to be considered on a case-by-case basis. Given increasing literature suggesting the risk of AS progression is directly related to baseline AVCS, the range of initial AVCS may guide the clinician regarding frequency of surveillance imaging and clinical follow-up [35].

From a technical perspective, AVCS is optimally collected on axial images as multiplanar reconstructions have been found to underestimate AVCS [35]. Care must also be taken to exclude calcification of LVOT, coronary arteries, mitral annulus, aorta, and aortic sinus as including such structures into the AVCS quantification may overestimate the true severity of AS [35,36].

Not infrequently, there may be discordance between AVCS and TTE grading of AS, with up to 25% of patients recording a discordant severity of AV stenosis on Doppler echo vs. AVCS (regardless of whether low-flow state is present) [41,84]. Although many of these cases may involve re-stratification of severity of AS by AVCS, on the rare occasion, there may be scenarios where AS is clearly severe by echocardiography, and yet have low AVCS. It has been hypothesized that such discordance may due to a predominant pathophysiology of valve fibrosis which precedes calcification, in particular in younger patients, female sex, or patients with BAV [43]. Such a scenario emphasizes the utility of AVCS as a complementary test to TTE evaluation of AS, and, therefore, to be assessed in the context of the overall clinical picture, rather than a standalone investigation.

Additionally, Wanchaitanawong et al. in 2023 highlighted another limitation of AVCS by CT in their evaluation of its use in rheumatic AV disease [78]. Analysis of 27 patients with rheumatic AV disease found no significant correlation with AVCS and TTE hemodynamics for AS severity. This was postulated to be a result of the pattern of calcium distribution, with a higher calcium load found on the aortic commissure compared to the valve leaflets (as is seen in TAV and BAV AS). These patients were also younger than typical TAV or BAV patients with AS, noting that age is a significant risk factor for the incidence of AV calcification [78].

Ultimately, this current review has evaluated the literature to date on this topic and, therefore, has no scope for reproducibility. It is an evolving field with ongoing research and increasing utilization of AVCS.

## 19. Discussion and Diagnostic Workflow

AS is a common and highly morbid condition with no current non-invasive therapeutic options to delay or prevent progression to a severity that may require valve replacement. The workhorse for detection, diagnosis, and assessment of severity of AS remains TTE. TTE has certain limitations, and occasionally assessment of AS severity may be challenging. AVCS has emerged as important complementary clinical tool for the diagnosis and grading of AS and has prognostic value.

AVCS has been validated to identify early AS, and generally correlates well with hemodynamic severity of AS. Sex-specific AVCS cut-offs for severe AS have been well established and subsequently integrated into European and American guidelines as a complementary investigation to TTE (ESC Guidelines: “very likely” severe AS ≥ 1600 AU for females and ≥3000 AU for males, “likely” severe AS ≥ 1200 AU for females, ≥2000 AU for males; AHA Guidelines ≥ 1300 AU for females, ≥2000 AU for males). These cut-offs have been clinically associated with prognosis and clinical events and may help guide the clinical decision for AVR, which may alleviate symptoms and restore prognosis when indicated.

AVCS has particular clinical utility in challenging patient subgroups including LFLG AS, pseudo-severe AS, paradoxical low-gradient AS or situations where there is overall clinical uncertainty. It is particularly valuable as a “tiebreaker” when there is discordance of TTE hemodynamic measurements regarding the severity of AS. A qualitative review of pattern of calcification may also provide value in the pre-TAVR work up by assisting anatomical planning.

## 20. Future Directions

Aortic valve calcification is easily discernible on all nongated CT chest scans performed for indications other than cardiac evaluation. As such, undiagnosed AS may be opportunistically identified on routine CT chest and trigger referral for further evaluation and diagnosis. With the rapid development of artificial intelligence, algorithms are being developed to opportunistically detect and quantify AV calcification on routine nongated CT scans.

Radiomics, which are quantifiable data extracted from medical imaging, represent a rapidly evolving field to increase accuracy and robustness of complex image pattern analysis, with early studies showing promise for both diagnostic and prognostic implications in CT angiography post-stroke [85]. This method has also been applied to CT chest images for CAC, with whole heart radiomics found to have higher diagnostic sensitivity for cardiovascular disease severity than traditional markers [86]. Although radiomics has primarily been utilized in oncology, early evaluation suggests significant potential for more accurate and efficient diagnosis of AV calcification [87].

Despite a lack of previous success in this domain, current research is also being directed towards identification of novel therapies that may potentially slow the progression of aortic valve calcification, such as the EVOID-AS trial [88,89]. The emergence of therapeutics that may slow the progression of AS, coupled with early opportunistic detection as afforded by CT, may revolutionize the landscape of AS management, a disease which carries such a poor prognosis in the absence of valve replacement once it becomes severe.

## 21. Conclusions

AVCS is a valuable complementary diagnostic test in the evaluation and prognostication of AS. It is useful clinically in grading the severity of AS, particularly in patients with discordant hemodynamic data or in contexts where echocardiography may underestimate severity. AVCS may help re-stratify patients who may benefit from aortic valve replacement, a treatment which alleviates symptoms and the poor prognosis associated with severe AS.

## Figures and Tables

**Table 1:** Summary of American Heart Association (AHA) and European Society of Cardiology (ESC) current guidelines for diagnosis and grading of aortic stenosis (AS) [3,11].

Guideline	Symptoms	Grading	TTE Criteria	Treatment
**AHA** (**3**)	Asymptomatic	**At risk** - BAV - Congenital valve anomaly - Aortic sclerosis	PAV < 2.0 m/s	Routine surveillance
		**Progressive (mild to moderate)** - Includes rheumatic valve changes (commissural fusion) Normal LVEF ± early LV diastolic dysfunction	Mild: PAV 2.0–2.9 m/s MG < 20 mmHg	Routine surveillance
			Moderate: PAV 3.0–3.9 m/s MG 20–39 mmHg	SAVR if undergoing other cardiac surgery
		**Severe AS**	PAV ≥ 4.0 m/s or MG ≥ 40 mmHg AVA ≤ 1.0 cm^2^ (or ≤AVAi 0.6 cm^2^/m^2^) +/− systolic dysfunction (LVEF < 50%)	AVR if: - LVEF < 50% - Undergoing other cardiac surgery - PAV ≥ 5 m/s or PAV increase ≥ 0.3 m/s/year - BNP > 3× normal - Exercise-symptoms or drop in BP from baseline (>10 mmHg) - Progressive decline in LVEF to <60% on ≥3 serial TTE
	Symptomatic	**Severe AS:** High-gradient	PAV ≥ 4.0 m/s MG ≥ 40 mmHg AVA ≤ 1.0 cm^2^ (or ≤AVAi 0.6 cm^2^/m^2^)	AVR
		**Severe AS:** Low-flow, low-gradient LVEF < 50%	**PAV < 4.0 m/s** **MG < 40 mmHg** AVA ≤ 1.0 cm^2^ *DSE → PAV ≥ 4.0 m/s with AVA < 1.0 cm^2^*	AVR If LVEF > 50%: - AVR if AS is most likely cause of symptoms
		**Severe AS:** Low-gradient, low-flow Normal LVEF ≥ 50%	**PAV < 4.0 m/s** **MG < 40 mmHg** AVA ≤ 1.0 cm^2^ (or AVAi ≤ 0.6 cm^2^/m^2^) *Stroke volume index < 35 mL/m^2^*	AVR
**ESC** (**11**)	Asymptomatic	**Moderate AS**	PAV < 4.0 m/s MG < 40 mmHg AVA > 1.0 cm^2^	Routine surveillance AVR considered if undergoing CABG or other cardiac valve/ascending aorta surgery
		**Pseudo-severe AS**	**PAV < 4.0 m/s** **MG < 40 mmHg** AVA ≤ 1.0 cm^2^ *Stroke volume index < 35 mL/m^2^* LVEF < 50% *DSE → AVA increases to >1.0 cm^2^*	Routine surveillance
		**Severe AS:** Normal flow, normal gradient	PAV ≥ 4.0 m/s MG ≥ 40 mmHg AVA ≤ 1.0 cm^2^	AVR if: - LVEF < 50% - Positive exercise test (symptoms/drop in BP below baseline) AVR if LVEF > 55% and normal exercise test and: - PAV > 5 m/s or MG ≥ 60 mmHg - Severe calcification (AVCS) and PAV increase ≥ 0.3 m/s/year - BNP > 3× normal
		**Severe AS:** Low-flow, low-gradient	PAV ≥ 4.0 m/s **MG < 40 mmHg** AVA ≤ 1.0 cm^2^ *Stroke volume index < 35 mL/m^2^* LVEF < 50%	AVR if no other cause for LV dysfunction OR if symptoms or drop in systolic BP (>20 mmHg) on exercise testing AVR if LVEF > 55% and: - MG > 60 mmHg or PAV > 5 m/s - AVCS confirms severe - Markedly raised BNP (>3 times normal)
	Symptomatic	**Severe AS:** Normal flow, normal gradient	PAV ≥ 4.0 m/s MG ≥ 40 mmHg AVA ≤ 1.0 cm^2^	AVR
		**Severe AS:** Low-flow, low-gradient	PAV ≥ 4.0 m/s **MG < 40 mmHg** AVA ≤ 1.0 cm^2^ *Stroke volume index < 35 mL/m^2^* LVEF < 50%	AVR if: - Contractile reserve on DSE (excluding pseudosevere) - If NO contractile reserve on DSE → AVR if high AVCS - LVEF > 50%, consider AVR after confirmation of severe AS (including high AVCS) - If undergoing CABG

**Abbreviations:** Transthoracic echocardiogram (TTE); aortic valve area (AVA); aortic valve area indexed to body surface area (AVAi); mean gradient across aortic valve (MG); peak velocity across aortic valve (PAV); aortic valve replacement (AVR); surgical aortic valve replacement (SAVR); transcatheter aortic valve replacement (TAVR); left ventricular ejection fraction (LVEF); dobutamine stress echocardiography (DSE); coronary artery bypass grafting (CABG); B-type natriuretic peptide (BNP); aortic valve calcium score (AVCS); blood pressure (BP).

## Data Availability

Data is contained within the article.
